# Alum Addition Triggers Hypoxia in an Engineered Pit Lake

**DOI:** 10.3390/microorganisms10030510

**Published:** 2022-02-26

**Authors:** Gerdhard L. Jessen, Lin-Xing Chen, Jiro F. Mori, Tara E. Colenbrander Nelson, Gregory F. Slater, Matthew B. J. Lindsay, Jillian F. Banfield, Lesley A. Warren

**Affiliations:** 1 Instituto de Ciencias Marinas y Limnológicas, Universidad Austral de Chile, Valdivia 5090000, Chile; 2 Department of Civil and Mineral Engineering, University of Toronto, Toronto, ON M5S 1A4, Canada; morij@yokohama-cu.ac.jp (J.F.M.); tara.nelson@utoronto.ca (T.E.C.N.); 3 Department of Earth and Planetary Science, University of California, Berkeley, CA 94706, USA; linxingchen@berkeley.edu (L.-X.C.); jbanfield@berkeley.edu (J.F.B.); 4 Graduate School of Nanobioscience, Yokohama City University, Yokohama 236-0027, Japan; 5 School of Earth, Environment and Society, McMaster University, Hamilton, ON L8S 4L8, Canada; gslater@mcmaster.ca; 6 Department of Geological Sciences, University of Saskatchewan, Saskatoon, SK S7N 5E2, Canada; matt.lindsay@usask.ca

**Keywords:** pit lakes, oil sands, tailing reclamation, hydrocarbon mining, ecological succession, aquatic microbiology

## Abstract

Here, we examine the geobiological response to a whole-lake alum (aluminum sulfate) treatment (2016) of Base Mine Lake (BML), the first pilot-scale pit lake established in the Alberta oil sands region. The rationale for trialing this management amendment was based on its successful use to reduce internal phosphorus loading to eutrophying lakes. Modest increases in water cap epilimnetic oxygen concentrations, associated with increased Secchi depths and chlorophyll-a concentrations, were co-incident with anoxic waters immediately above the fluid fine tailings (FFT) layer post alum. Decreased water cap nitrate and detectable sulfide concentrations, as well as increased hypolimnetic phospholipid fatty acid abundances, signaled greater anaerobic heterotrophic activity. Shifts in microbial community to groups associated with greater organic carbon degradation (i.e., SAR11-LD12 subclade) and the SRB group Desulfuromonodales emerged post alum and the loss of specialist groups associated with carbon-limited, ammonia-rich restricted niches (i.e., MBAE14) also occurred. Alum treatment resulted in additional oxygen consumption associated with increased autochthonous carbon production, watercap anoxia and sulfide generation, which further exacerbate oxygen consumption associated with on-going FFT mobilized reductants. The results illustrate the importance of understanding the broader biogeochemical implications of adaptive management interventions to avoid unanticipated outcomes that pose greater risks and improve tailings reclamation for oil sands operations and, more broadly, the global mining sector.

## 1. Introduction

Large volumes of fluid fine tailings (FFT) produced during bitumen extraction create significant challenges for mine reclamation in the Alberta oil sands region (AOSR) of northern Alberta, Canada. These tailings are initially composed of process-affected water (65–75 wt.%), fine-grained solids (25–35 wt.%), and residual petroleum hydrocarbons (<5 wt.%). Oil sands process-affected water (OSPW) is characterized by elevated concentrations of dissolved salts, NH_4_^+^, ∑H_2_S, and detectable concentrations of various organics derived from oil sand deposits and the bitumen extraction process [1,2]. Elevated CH_4_ concentrations within FFT deposits result from the methanogenic degradation of fugitive diluent hydrocarbons and, to a lesser extent, residual bitumen [3,4,5]. Post-depositional settlement and dewatering produces a free water layer that gradually deepens with time. Slow pore water release coupled with growing production mean the total FFT inventory has grown to over 1.2 billion m^3^ across the AOSR [6].

Regulations aimed at promoting progressive reclamation and curbing inventory growth have accelerated the development of various FFT treatment and reclamation strategies [6,7,8]. Pit lakes (PL) are a key reclamation strategy being developed at oil sands mines [7]. These mine closure landforms, which are established in exhausted mine pits, are being developed for the reclamation of FFT, treated FFT, and OSPW [9,10,11]. Water-capped tailings technology (WCCT), i.e., PL, involves permanent FFT storage below a relatively shallow water cap initially dominated by OSPW [6]. Although water quality improvements driven by freshwater inputs and in situ biogeochemical processes are anticipated, many uncertainties concerning the long-term viability WCTT require investigation. In particular, PL need to maintain an oxygenated zone that can support biota and ecological function [2,12].

Extensive research focused on oil sands tailings ponds suggests that suspended fine-grained solids and oxygen-consuming constituents (OCC), including NH_4_^+^, ∑H_2_S, CH_4_ and other organics, derived from FFT could inhibit the development of a persistent oxic zone within the upper water cap of PL supporting WCTT. Permanent anoxia within the free water layer of these tailings ponds results from extensive O_2_ consumption associated with high levels of microbial activity [3]. High turbidity resulting from the slow settlement of fine-grained FFT-derived particles and/or suspended solids contributions from oil sands process water (OSPW) within the water cap may also limit primary production and thus biological O_2_ production [13]. Research into these and other aspects of WCCT has largely focused on Base Mine Lake (BML), which was the first full-scale PL developed in the AOSR.

Early developmental stage investigation of the BML summer thermally stratified water cap three years post commissioning [2,12,14] identified that O_2_ concentrations decreased with depth and were always less than 100% saturated (surface August epilimnetic upper water cap oxygen concentrations averaged ~156 µM in 2015 and ~188 µM in 2016 [12] in the top portion of the water cap during peak summer stratification periods (i.e., where photosynthesis would be most likely to occur)). These results are consistent with other studies that suggested photosynthetic activity was impaired in this early stage due to a lack of water clarity [15] and/or associated with periodic lake surface hydrocarbon sheens [16]. However, both Risacher et al. [12] and Arriaga et al. [2] revealed low but persistent O_2_ concentrations within ~2–3 m of the FFT water interface (FWI), i.e., <10 µM or 0.25 mg L^−1^ throughout the summer; oxygen consumption driven by mobilized reductants from the underlying FFT was not sufficient to cause anoxia.

In the fall of 2016, a whole lake alum (aluminum sulfate) addition trial was implemented to improve the clarity of the BML water cap, in aid of promoting greater primary productivity and increased oxygen concentrations [17]. Alum forms Al hydroxide flocs upon contact with water that can collect suspended particulates and sorb dissolved compounds as they settle out of the water column [18]. The rationale for trialing this management amendment was based on its successful use around the world to reduce internal phosphorus loading to eutrophying lakes and to clarify drinking water in water treatment plants, resulting in increased oxygen concentrations, and to clarify drinking water in water treatment plants [19,20,21,22,23,24].

However, the proposed BML alum addition was also likely to be a major disturbance impacting the water cap and potentially the FFT water interface (FWI) microbial communities, either through the stripping of water cap microbes associated with alum floc particle settling out, and/or changes to water cap ecological niches, which could affect BML water cap biogeochemical cycling and oxygen outcomes. Disturbances, whether alum amendments to soils or eutrophying lakes [25,26], or other perturbations (i.e., changes in soil use, or altered wet–dry cycles simulating climate change effects; [27,28]) have been shown to change microbial community structure, function, and thus ecological outcomes. For instance, soil microbial communities have been shown in both field and in micro/mesocosm experiments to exhibit long-term changes in their structure following a disturbance [29,30].

Thus, investigation of the effects of alum addition on the microbial ecology and biogeochemical cycling in engineered ecosystems such as PL, which differ in their biogeochemical characteristics compared with more natural contexts, is vital to understanding the key processes affecting water cap oxygen outcomes. As a further 23 pit lakes are currently planned in the AOSR [2], this whole lake alum amendment provides a unique opportunity to inform the development of models that forecast processes and outcomes in these engineered contexts, as well as contributing to the understanding of aquatic systems undergoing disturbance through the characterization of microbial adaptation and system responses to this major perturbation. Here, our objectives were to identify changes in: (1) microbial community structure and function, (2) biogeochemical cycling, and (3) observed water cap O_2_ concentrations, through integrated geochemical and microbiological analyses of peak summer stratified, depth-dependent water samples collected pre and post alum addition.

## 2. Materials and Methods

### 2.1. Site Description

**Figure 1:**
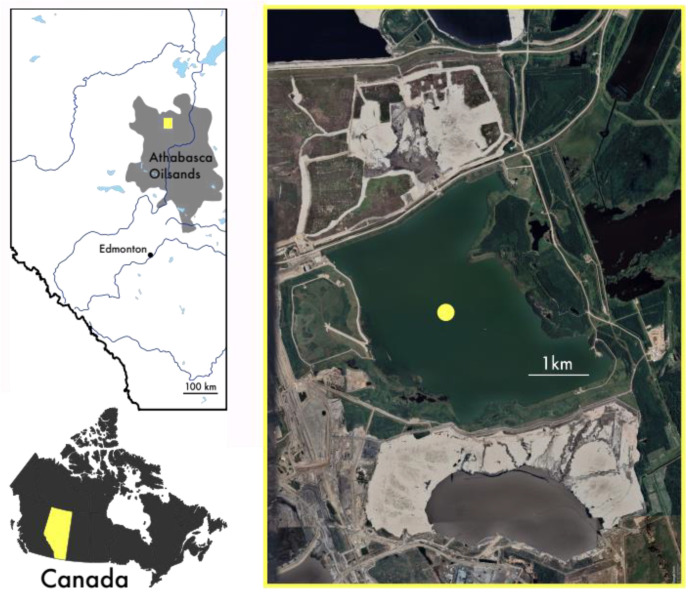
Location of Athabasca Oilsands region (grey) in Alberta, Canada. Aerial photo of Base Mine Lake (right panel) depicting sampling station (yellow dot).

BML has now been described in several studies [2,12,15,16,31]. Briefly, BML (57°1′ N, 111°37′ W, elevation 308 m, surface area 7.8 km^2^) was built in a mined-out oil sands pit at the Syncrude Canada Mildred Lake mine near Fort McMurray, AB Canada (Figure 1). Originally BML was a tailings impoundment that received FFT inputs from 1994 to 2012. The thickness of the FFT in 2012 was 45 m with a 3–5 m dominantly OSPW water cap on top. In November 2012, BML was disconnected from tailings operations, and freshwater from a nearby natural lake was added to increase the water depth to a level sufficient to prevent wind-wave-induced erosion of the underlying FFT material. Since 2012, with the addition of the freshwater and the on-going dewatering and consolidation of the underlying FFT, BML’s water cap has increased in depth from ~8–9 m in 2012 to 10–11 m by 2018. In the fall of 2016, a whole lake addition of alum (3CaAl_2_(SO_4_)_3_14.3H_2_O) to the BML water cap was made to promote clarity and encourage consolidation of the suspended solids, contributing to high turbidity within the water cap [17].

### 2.2. Sampling Strategy

Sampling campaigns were carried out on BML for three summers during maximum thermal stratification (August 2016–August 2018). Methods and sampling protocols have been described elsewhere [2,12,14]. Briefly, a physicochemical profile of the BML water cap (temperature, pH, oxygen, specific conductivity, oxidation reduction potential (ORP)) was conducted using a YSI Professional Plus 6-Series Sonde (YSI Incorporated, Yellow Springs, OH, USA). Water samples were collected for both geochemical and microbiological characterization. Bulk water was retrieved in a 6.2 L Van Dorn bottle (Wilco, model Beta^®^, Wildco, Yulee, FL, USA), while FFT was sampled using a custom-built fixed interval sampler as described in Mori et al. [14] and Dompierre et al. [32].

### 2.3. Geochemical Analyses

CH_4_, and concentrations of dissolved (<0.45 µm) aqueous species of NH_4_^+^, NO_2_^−^, NO_3_^−^, ΣH_2_S, and SO_4_^2−^, were analyzed as described in Risacher et al. [13] and Arriaga et al. [2]. Briefly, [∑H_2_S] was analyzed via the methylene blue method directly after sampling using a HACH portable spectrophotometer (Hach DR/2800 spectrophotometer, HACH Company, Loveland, CO, USA). Dissolved methane was analyzed on an SRI GC (model 8610C, SRI Instruments, Torrance, CA, USA) equipped with a 1 m silica gel column and a flame ionization detector. SO_4_^2−^ and nitrogen species were analyzed spectrophotometrically following HACH DR/2800 spectrophotometer standard methods (HACH Company, Loveland, CO, USA). Bulk carbon, i.e., total organic carbon (TOC) and dissolved organic carbon (DOC), was analyzed as described in Whaley-Martin et al. [33]. In summary, frozen samples were thawed, filtered (for DOC only) and analyzed using a Shimadzu TOC-L (Shimadzu Precision Instruments, Kyoto, Japan). Organic carbon values were determined by subtracting the concentration of inorganic carbon from the total carbon values for that filter fraction (unfiltered, or 0.45 µm filtered).

### 2.4. DNA Extraction and Quantification

Water and FFT cells were retrieved by filtering ~1.5 L water through 0.22 μm Rapid-Flow sterile disposable filters (Thermo Fisher Scientific (Waltham, MA, USA)) and from ca. 50 g wet FFT, respectively, and stored at −20 °C until DNA was extracted. DNA was extracted and purified from water column samples using the DNeasy PowerWater DNA Isolation Kit (Qiagen, Hilden, Germany), while ca. 0.5 g of each FFT sample was extracted using the FastDNA Spin Kit for Soil (MP Biomedical, Irvine, CA, USA). DNA concentration and quality were confirmed with a Qubit 2.0 Fluorometer (Thermo Fisher Scientific, Waltham, MA, USA) using a Qubit High-Sensitivity dsDNA Assay Kit (Thermo Fisher Scientific, Waltham, MA, USA), and DNA quality was assessed using an Epoch Microplate Spectrophotometer with Take3 plate (BioTek, Winooski, VT, USA).

### 2.5. Amplicon Sequencing

The same set of primers and the variable region of the 16S rRNA gene were used to characterize the microbial community structure in both water and FFT samples. The primer set 515f (5′-GTGYCAGCMGCCGCGGTAA-3′) and 806r (5′-GGACTACNVGGGTWTCTAAT-3′) was used to amplify the V4 region of the bacterial and archaeal 16S rRNA gene [34] following standard protocols of the Earth Microbiome Project [35,36]. In short, the PCR reaction protocol was conducted as follows: 50 ng of DNA was denatured at 98 °C for 5 min, followed by 35 cycles of denaturing at 98 °C for 30 s, annealing at 50 °C for 30 s and extension at 72 °C for 30 s, followed by final extension at 72 °C for 10 min [34]. Gel electrophoresis was used to check DNA products and positive amplicons were sequenced using the Illumina MiSeq platform. Water cap microbial DNA was sequenced at Farncombe Metagenomics Facility at McMaster University (Hamilton, ON, Canada) while DNA was extracted from FFT at the University of Calgary (Calgary, AB, Canada). The SequalPrep normalization kit (Thermo Fisher Scientific, Waltham, MA, USA) was used for amplicon normalization and sequenced with the Illumina MiSeq platform. Sequence reads were filtered and trimmed using Cutadapt (minimum quality score of 30 and a minimum read length 100 bp) [37]. Sequence variants were resolved using DADA2 [38] and merged to combine all information from separate Illumina runs. Chimeras and bimeras were removed along with sequences belonging to Eukaryota, chloroplasts and mitochondria. Taxonomy was assigned using the SILVA database version 132 [39]. Water column and FFT 16S rRNA gene amplicon sequences are deposited at NCBI under Bioproject PRJNA552483.

### 2.6. Statistical Analyses

Patterns of alpha and beta diversity were calculated from the relative abundance of sequence variants, whereas changes in community composition were explored using non-metric multidimensional scaling (NMDS; Bray–Curtis dissimilarities). Analysis of similarity (ANOSIM) was used to test differences between years and *p*-values corrected using Bonferroni’s correction. Multivariate Redundancy Analyses (RDA) were performed to investigate the community response to the environment. Prior to the analyses, the complete physicochemical and geochemical characterization of the system was standardized (Z-scored) and checked for multicollinearity. After stepwise variable selection (based on the variance inflation factor; VIF), aluminum addition, temperature, oxygen, methane, nitrite and nitrate were retained. Multiple partial RDAs and variation partitioning (VP) analysis were used to assess significance and percentage of contribution to microbial variability. All statistical analyses were conducted following Buttigieg and Ramette [40] and Ramette [41] and using vegan R scripts [42] (version 3.6.2; www.R-project.org, accessed on 12 December 2019).

## 3. Results and Discussion

### 3.1. Pre- and Post-Alum BML Water Cap Physico-Chemistry and Geochemistry

**Figure 2:**
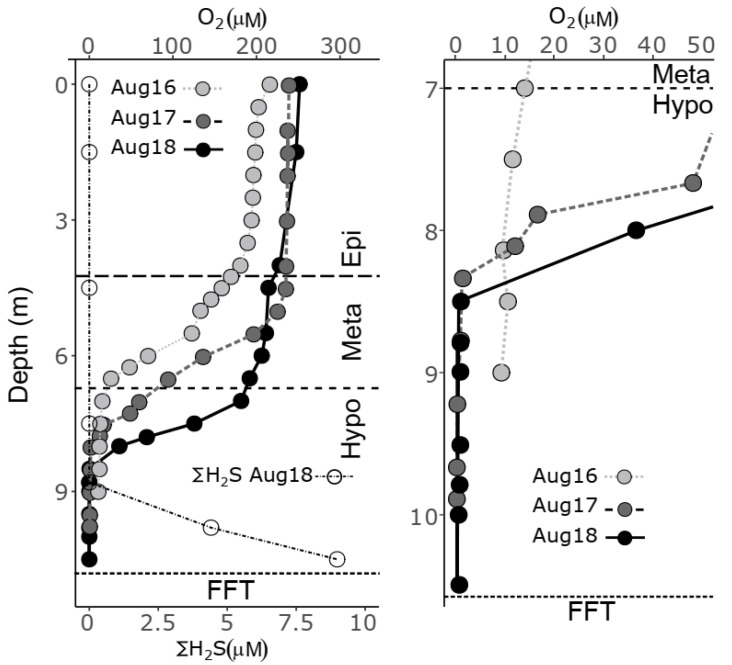
Water column (**left**) and bottom water (**right**) oxygen and sulfide concentrations for August 2016–2018 at BML.

No observable changes in thermal profiles were measured comparing pre-alum August 2016 thermal zonation to post-alum BML water cap stratification in August 2017 and August 2018 (Figure 2). For all three years, the August BML water cap exhibited maximum summer stratification and evidenced similar epilimnetic (0–4 m), metalimnetic (4–6.5 m) and hypolimnetic (6.5–10 m, FFT water interface (FWI)) zonation. In contrast, a modest increase (11–18%) in maximum epilimnetic oxygen concentrations occurred post alum, increasing from 214 µM in August of 2016, pre alum, to 238 µM in 2017 and to 251 µM in 2018 (Figure 2). However, lower hypolimnetic oxygen concentrations of the water above and immediately adjacent to the FWI, decreased from severe hypoxic ~10 µM in 2016 pre alum, to anoxic over the lowest 1.75 m above FWI in 2017 and euxinic in 2018 post alum ([∑H_2_S] up to 9 µM, Figure 2, Table 1).

While epilimnetic oxygen concentrations increased 17% between 2016 and 2018 (Table 2), carbon concentrations, whether total carbon (TC: filtered (TCF) or unfiltered (TCU)), TOC or DOC, evidenced successive decreases in both 2017 and 2018 post alum addition, whether considering averaged values over the entire water column or the hypolimnetic zone (Table 2). The relative percent (∆%) decrease in absolute concentrations ranged from 25% for both averaged entire water cap or hypolimnion TCF and TCU, to 59–60% and 66–68% for averaged entire water cap or hypolimnion DOC and TOC concentrations (Table 2) from 2016 to 2018. In addition, the relative proportion of the total carbon occurring as either DOC or TOC also decreased for the averaged entire water cap or the hypolimnion by 23%, 28%, 31% and 36%, respectively. The maximum dissolved methane concentrations observed at the FWI also decreased substantially (82–83%) from 161 µM in 2016 to 29 µM (2017) and 27 µM (2018; Table 1) post alum with upper water cap concentrations remaining low (<1 µM) for all years. FWI ammonia concentrations also decreased post alum from a maximum observed in 2016 of ~50 µM to ~40 µM in 2017 and ~29 µM in 2018 (Table 1); however, upper water cap ammonia concentrations increased from <10 µM (2016), to <20 µM (2017), to <30 µM (2018; Table 1), while no observable changes in organic phosphorus were measured (2.27 ± 0.5 µg L^−1^, Table S1). The divergent responses of these two previously identified important OCC were shown to dominantly influence BML water cap oxygen concentrations pre alum [2,13], suggesting the post alum development of lower BML water cap euxinia reflects a more complex set of processes.

Nitrate concentrations were highest in the upper waters in both 2016 (pre alum, ~30–40 µM) and 2017 (1st year post alum, 50–55 µM), decreasing towards the FWI (Table 1). However, while nitrate, along with oxygen, persisted to the FWI in 2016, it was below detection in the bottom anoxic meter of the BML water cap in 2017 and was completely non detectable throughout the water cap in 2018 (Table 1), indicating that a lack of nitrification and greater nitrate consumption alongside oxygen consumption were occurring and increasing year over year for the two years investigated, post alum. Concentrations of sulfate were orders of magnitude higher (2–3 mM) compared with other constituents throughout the water cap across all years (Table 1). Mean August water cap sulfate concentrations increased 27% from 2045 µM (2016) to 2603 µM in 2017, consistent with the addition of sulfate from alum. However, sulfate concentrations decreased in 2018 to pre-alum 2016 levels, averaging 2072 µM. This result suggests that beyond the ongoing settling of the FFT, anoxia enabled SRB presence and activity within the water cap, consistent with greater reductive metabolism occurring in BML post alum (Table 1). Furthermore, for the first time in 2018, detectable ∑H_2_S occurred in the bottom anoxic waters of BML (9 µM; Table 1) providing evidence of SRB activity. These geochemical results collectively indicate changes in microbial redox dynamics with greater overall reductive processes occurring in BML post alum addition.

### 3.2. FFT Porewater Chemistry

Porewater chemistry for the upper 0.5 m of FFT is summarized for August 2016 (pre alum) and July 2017 (post alum) in Table 1 and in more detail in Mori et al. [14]. Briefly, highly anaerobic conditions persisted in both years, as shown by high dissolved concentrations of ammonia (>600 µM), methane (>1200 µM), and ∑H_2_S (>10 µM) that increased with depth in the upper FFT (Table 1, [14,43]). Sulfate concentrations decreased substantially (<100 µM) over the same depth, representing a 90% decrease relative to the water cap [14,43]. Low sulfate concentrations coupled with elevated ∑H_2_S concentrations suggest sulfate reduction activity occurring in the upper FFT, consistent with the apparent depletion of other, higher energy electron acceptors (i.e., nitrate, ferric iron) within the FFT porewater. Porewater DOC concentrations in the upper FFT increased slightly to >6 µM with depth below the FWI.

Porewater CH_4_, NH_4_^+^ and ∑H_2_S concentrations were generally consistent between 2016 and 2017 (Table 1), suggesting the effects of alum treatment on water chemistry did not extend below the FWI. These results are consistent with previous findings that the FFT microbial community structure exhibited greater variation with depth than over time [14,43].

### 3.3. Shifts in Microbial Community Structure through Time

**Figure 3:**
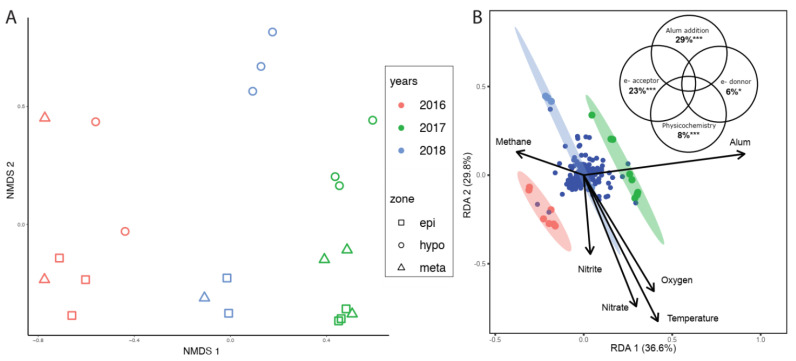
(**A**) Non-metric multidimensional scaling (NMDS) plot of samples at sequence variants level (based on Bray–Curtis dissimilarity from Illumina sequencing of 16S rRNA gene amplicons), depicting differences between microbial community structure among years (August 2016–2018) and stratifications zones at Base Mine Lake (stress = 0.11, ANOSIM; R = 0.8–0.9, *p* < 0.001). (**B**) RDA ordination depicting microbial diversity (based on sequence variants) in relation to significant aqueous physico-chemistry and geochemistry. The Venn diagram shows the significant effect (%) of physico-chemistry (temperature), e- acceptor (oxygen, nitrite and nitrate), e- donor (methane) and alum (AlSO_4_^2−^) treatment on the bacterial diversity at BML (*p* < 0.001). *** *p* < 0.001, * *p* < 0.03.

Evident changes in BML water cap microbial community structure occurred post alum addition (Figure 3A,B, Table S2), consistent with the collective observed geochemical shifts, suggesting greater e- acceptor consumption and the production of reduced metabolites. In 2017 and 2018, BML water cap microbial communities significantly increased in richness (i.e., a greater number of species, F(2,22) = 3.6, *p* < 0.05; Tukey post hoc test, *p* < 0.05); however, diversity significantly decreased (i.e., integrating both the number of species and the abundance of individuals within each species, (F(2,22) = 9.1, *p* < 0.01; Tukey post hoc test, *p* < 0.01)) suggesting the possible emergence of pioneer species after the alum addition. As Rönicke et al. [26] identified, studies that report on the long-term consequences of alum addition to the ecology of lakes are rare, despite the use of alum for the treatment of eutrophying systems for over 40 years. However, Lin et al. [44] identified no significant difference in rhizosphere community composition, but a significant difference in the abundance of the major taxa associated in experiments assessing Al addition to soil. Wang et al. [27] also showed abundance changes in carbon-fixing bacterial taxa in wetland soils associated with disturbance and reclamation, while Zhang et al. [28] identified that compositional changes in microbial communities occurred in soils associated with different tillage regimes, while diversity remained unchanged. In this latter study, they attributed the differences in abundances to changes observed in soil organic carbon fractions.

Losses and gains in species diversity affect the ecological stability and sustainability of ecosystem services. Decreased diversity has been suggested to decrease ecological stability and a community’s resistance to environmental change and rates of recovery from further disturbances [45]. A nonmetric multidimensional scaling (NMDS) ordination plot (based on Bray–Curtis distance matrix of amplicon sequence variants (ASVs)) indicated that these alum-associated BML microbial community differences were significant across all years, i.e., the 2nd year post alum, 2018, was also significantly different from 2017 (ANOSIM, Bonferroni corrected, *p* < 0.005, Figure 3A). Indeed, alum addition was raised as the main factor structuring the microbial community, followed by oxygen, nitrite and nitrate (*p* < 0.001, Figure 3B). This is consistent with a rapid response in the microbial community to the initial ecosystem perturbation and an on-going differentiation of the BML water cap microbial communities two years post alum addition.

**Figure 4:**
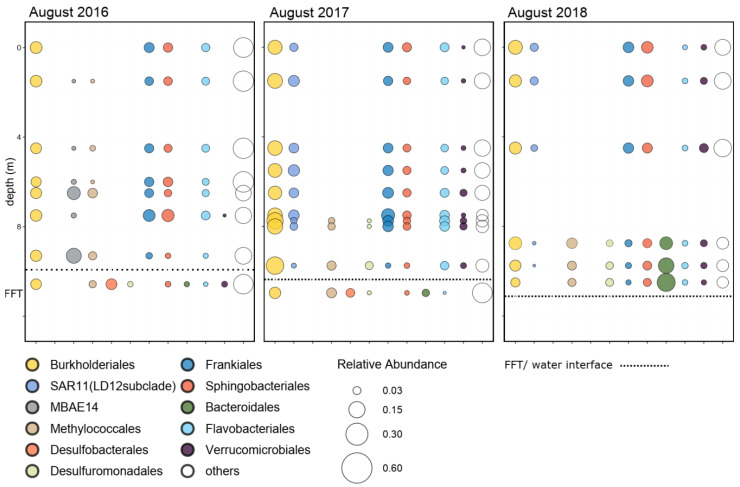
Relative abundance of bacterial dominant orders, detected by Illumina sequencing of 16S rRNA gene amplicons, for the Base Mine Lake water cap (summer 2016–2018) and FFT water interface (2016, 2017).

Microbial redox and successional shifts are linked to shifts in diagenetic transformations, suggesting that changes in the quantity of specifically labile organic carbon occurred post alum. While decreases in TOC and DOC concentration were noted, the relatively unchanged dominating abundance and distribution of Burkholderiales, both in the water column and FFT, is consistent with the dominance of refractory carbon materials, as members are known for their effective utilization of numerous recalcitrant organic compounds [46] (Figure 4). In contrast, MBAE14, present in 2016 pre alum, vanished, while SAR11 (LD12 subclade) appeared in high abundance after alum addition (Figure 4). Members of the novel MBAE14 group inhabit a narrow habitat of ammonia-rich, oxygen- and labile organic matter-limited conditions that characterized BML up to 2016 [14]. In contrast, the aerobic chemo-organotrophic lifestyle of the SAR11 (LD12 subclade) [47] suggests their emergence post alum is linked to an increase in the quantity of higher quality (i.e., more accessible) organic matter. The occurrence of anoxia in the bottom waters post alum, along with the decreased concentrations of nitrate and sulfate and increased ammonia concentration in the BML water cap by 2018 especially (Table 1), are collectively consistent with greater heterotrophic activity. These results suggest that an important effect of the alum addition was to stimulate the generation of autochthonous organic carbon, i.e., phytoplankton, that could support greater heterotrophic activity. Similarly, Flavobacteriales (mainly comprising the genus Flavobacterium) were also ubiquitous across all three years, suggesting that this group, with its metabolic versatility and copiotrophic characteristics [48], may also adapt well to varying organic matter quality and availability and oxygen conditions.

Further shifts reflect rapid responses by the microbial community to post alum changes in important e- donors and e- acceptors, which variably limited or enabled the required ecological niches. Taxa belonging to the order Frankiales, which are known to include those capable of nitrate reduction [49], were restricted to the water column and decreased in abundance in 2018, consistent with the marked absence of detectable nitrate concentrations in the 2018 BML water column (Figure 4). Groups related to methanotrophic isolates [50], such as Methylococcales, decreased in abundance and distribution, with apparent restriction to the bottom waters only (Table 1, Figure 4) coincident with the observed decrease in water cap dissolved methane concentrations post alum (Table 1). In contrast, Bacteroidales, restricted to the reducing conditions associated with FFT, pre alum, colonized the water column when anoxic/euxinic conditions occurred post alum, indicating its ability to migrate into the water cap once oxygen was depleted (Figure 4, Table 1). Similarly, Desulfuromonadales also migrated out of the surficial FFT, extending its presence into the newly anoxic, sulfate-rich bottom waters that occurred post alum (Figure 4). Mainly comprising Geobacter affiliates, their migration into these anoxic waters is consistent with the demonstrated functional capabilities of dissimilatory metal and sulfur reduction in hydrocarbon-contaminated sediments using the wide range of monomers shown for isolates of this genus [51]. Even though originally considered strict anaerobes, members of this group are also capable of microaerobic growth [52], enabling them to extend beyond the anoxic into hypoxic BML bottom waters after the addition of alum (Table 1). In contrast, Desulfobacterales was confined exclusively to the FFT, which may reflect its preference for fermentation products via sulfate reduction [53], which may be limited in the newly anoxic waters, and/or their potentially stricter anaerobic requirements. Interestingly, known relatives are capable of benzene degradation, i.e., more recalcitrant organic carbon compounds, under methanogenic conditions [54], suggesting that this order may play an important role in the later development of the lake. The results provided here are some of the first to document changes in microbial communities, post alum addition, and illuminate the rapidity with which microorganisms will respond to perturbations that change environmental characteristics.

### 3.4. Improved Clarity and Biogeochemical Consequences in BML

While absolute concentrations of TOC/DOC would classify BML as a meso-eutrophic system across all three sampling years, organic carbon pools in BML are largely dominated by recalcitrant, complex organic carbon moieties associated with the bitumen extraction of oil sands [5], which would overwhelm the likely small, but important increases in autochthonous organic carbon that were suggested to occur by the collective geochemical and microbial community results, post alum. While all carbon concentrations decreased post alum, suggesting physical stripping out had occurred; the relative proportion of organic to total carbon decreased post alum across the water cap, with the relative % decrease being the highest for hypolimnetic TOC and DOC relative to TC concentrations (36% and 31%, respectively; Table 2). These results suggest that greater degradation and thus decreased organic carbon occurred post alum addition. The stimulation of autochthonous organic carbon is supported by the increased Secchi disk depths (Figure S1), epilimnetic oxygen concentrations, Chla concentrations and periphyton abundance observed in 2017 and 2018 (Figure S2). These new autochthonous food web sources of more degradable organic carbon would, in turn, support greater rates of decomposition and heterotrophic microbial biomass. For instance, members of Verrucomicrobiales became dominant after the alum addition, consistent with their ability to degrade chitin [55], an important constituent of zooplankton, such as the *Daphnia pulex* exoskeleton, animals that also increased in abundance post alum (data not shown).

Changes in phospholipid fatty acid (PLFA) concentrations and profiles consistent with this scenario also occurred, as described in more detail in Slater et al. [56]. Specifically higher PLFA concentrations and increased autotrophic PLFA abundance (i.e., C18:1 unsaturated, polyunsaturated), as well as δ^13^C values of PLFA consistent with autotrophy, collectively indicate the establishment of an epilimnetic phototrophic microbial community post alum. Transport of organic biomass to the hypolimnion, hypothesized here to drive the initiation of anoxic conditions observed post alum, is reflected in the recognizable signature of the phototrophic (epilimnetic) communities (increased C18:1 and polyunsaturated PLFA) in hypolimnetic samples post alum.

Furthermore, 2018 PLFA distributions continued to indicate the presence of an epilimnetic phototrophic community, though at lower abundances than in 2017, as well as the downward transport of this algal biomass into the hypolimnion (higher phototrophic PLFA abundance). However, in contrast to 2017, in 2018, there was a notable increase in branched and cyclic PLFA, particularly in the hypolimnion. Branched PLFA are generally associated with heterotrophic microbial communities, suggesting the greater establishment of heterotrophy in the bottom waters of BML by 2018. Stable isotopic compositions (δ^13^C) of PLFA in 2018 showed some of the largest changes over the four-year interval studied in Slater et al. [56]. Most notably, there was a convergence of epilimnetic δ^13^C of PLFA in August 2018 to that observed in May 2017, consistent with the establishment of a phototrophic community two years post alum addition. The observed negative shift in δ^13^C of PLFA in the 2018 August hypolimnion is consistent with the greater contributions of anaerobic metabolism to the microbial PLFA pool.

## 4. Conclusions

**Figure 5:**
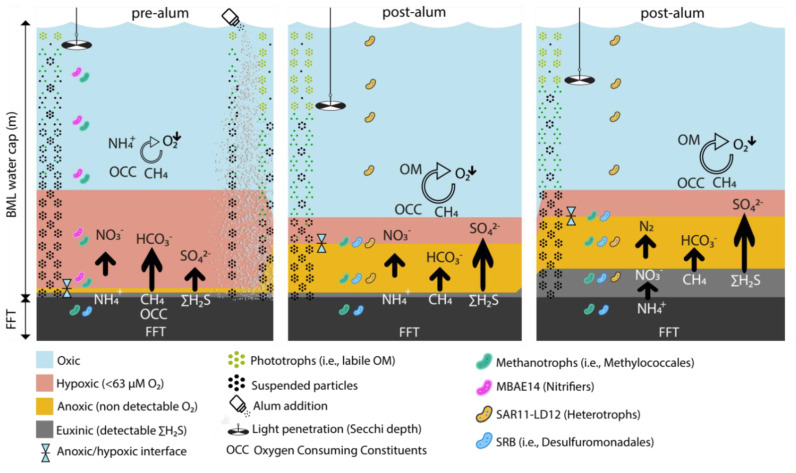
Schematic model depicting the biogeochemical trajectory impairing oxygen concentrations in BML resulting from alum addition. Prior to alum addition (2016), the major oxygen-consuming constituents were mobilized reductants from the underlying FFT layer. Alum addition in the fall of 2016 removed particles from the water cap, improving clarity and enabling greater primary production in 2017 and 2018. Increased oxygen consumption driven by the degradation of more abundant biomass affected oxygen status post alum with increased anoxia (2017, 2018), the upward migration of the anoxic–hypoxic interface and the generation of sulfide directly within the water cap.

The whole lake alum amendment, trialed in BML to clarify the lake through tailings sequestration, achieved this objective, as observed in increased Secchi disk depths in 2017 and 2018. It also resulted in increased autochthonous carbon production, evidenced by increased Chla, periphyton biomass and epilimnetic oxygen concentrations in the two August sampling periods post alum addition. However, hypolimnetic anoxia also occurred, contrasting with the observed oxygen outcomes of alum treatment in eutrophying lakes [24]. The results observed in BML reflect the significant differences in the sources of oxygen-consuming constituents (OCC) in these two types of systems. The major oxygen-consuming process in eutrophic lakes is the decomposition of abundant biomass material generated by excess nutrient loads. Thus, in a eutrophic lake, alum increases the overall oxygen concentrations in the water column by reducing nutrients, biomass generation (major reductant) and oxygen consumption. Prior to alum addition, BML water cap oxygen consumption was primarily driven by the mobilization of reductants from the underlying FFT layer, and its organic carbon pool was dominated by recalcitrant organic moieties, i.e., easily degraded organic carbon was highly limited and its contribution to overall oxygen consumption minimal (Figure 5).

The increased oxygen consumption associated with aerobic degradation of this new biomass source induced anoxia and enabled anaerobic biogeochemical cycling directly within the BML water cap post alum. Rapid interlinked structural and functional changes in the microbial community, important to biogeochemical cycling and overall oxygen status of BML, occurred in response (Figure 5). Specifically, microbial groups associated with labile organic carbon processing appeared (i.e., SAR11-LD12 subclade); specialist groups such as MBAE14, associated with carbon-limited, ammonia-rich restricted niches that existed in BML prior to alum addition [14], disappeared; and the SRB group Desulfuromonodales appeared (Figure 4 and Figure 5).

Enabling SRB activity directly within the water cap—where sulfate concentrations are high (~2 mM)—is likely to exacerbate oxygen consumption, as the oxygen-driven oxidation of sulfide, abiotically and/or biotically, will be rapid under BML geochemical conditions. Ultimately, the developmental trajectory of BML water cap oxygen status—i.e., the spatial and temporal extent of anoxia—will now reflect the balance between oxygen inputs associated with epilimnetic primary production and physical mixing processes, and this expanded set of oxygen consumption processes of biomass degradation, sulfide oxidation and on-going FFT reductant mobilization (Figure 5). The results here identify the need to fully understand the biogeochemical processes affecting oxygen concentrations and distribution in these engineered systems, such that the effects of potential treatment strategies can be accurately predicted. More broadly, these results contribute to our understanding of perturbation effects on microbial community structure and function in aquatic systems.

## Figures and Tables

**Table 1:** Aqueous and FFT physico-and geochemical characterization of BML.

Year	Depth [m]	Oxygen (µM)	Temp (C)	pH	EC (mScm^−1^)	TOC (mgL^−1^)	DOC (mgL^−1^)	Methane (µM)	Ammonia (µM)	Nitrite (µM)	Nitrate (µM)	Sulfate (µM)	Sulfide (µM)
2016	0.00	214.2	20.8	8.2	2.82	99.9	87.7	0.6	9.9	1.5	38.8	2026.7	0.0
2016	0.50	200.8	20.7	8.3	2.82								
2016	1.00	197.7	20.5	8.3	2.82								
2016	1.50	196.9	20.4	8.3	2.82	58.8	57.5	0.5	8.6	2.0	36.0	2212.1	0.0
2016	2.00	194.7	20.3	8.3	2.82								
2016	2.50	194.0	20.3	8.3	2.82								
2016	3.00	192.5	20.2	8.3	2.82								
2016	3.50	187.7	20.0	8.3	2.82								
2016	4.00	179.0	19.8	8.3	2.82								
2016	4.25	167.9	19.5	8.3	2.82				6.7	1.9	34.0	2280.9	0.0
2016	4.50	156.7	19.3	8.2	2.82	58.4	58.3	0.4	10.4	2.3	39.4	1709.5	0.0
2016	4.75	144.0	19.1	8.2	2.82				8.1	1.4	39.4	2314.4	0.0
2016	5.00	131.3	18.9	8.2	2.82								
2016	5.50	120.9	18.6	8.2	2.82			0.7	6.0	1.1	39.0	2333.8	0.0
2016	6.00	68.6	17.5	8.1	2.84	60.7	60.9	0.6	6.2	6.2	47.2	2115.4	0.0
2016	6.25	46.5	17.0	8.1	2.85				7.5	1.6	31.4	1869.1	0.0
2016	6.50	24.3	16.6	8.0	2.87			0.2	15.9	10.3	45.7	1824.1	0.0
2016	7.00	13.9	15.6	8.0	2.88	61.0	59.0	11.1	22.1	5.0	43.4	2039.3	0.0
2016	7.50	11.4	15.0	7.9	2.89			9.5	16.8	5.8	34.2	1802.5	0.0
2016	8.00	10.5	14.5	7.9	2.90	94.5	87.5	39.0	27.9	4.7	12.8	2007.5	0.0
2016	8.50	10.5	14.3	7.9	2.91								
2016	9.00	9.1	14.1	7.9	2.89			126.3	49.7	0.8	15.5	2049.8	0.0
2016	(FFT)−0.05	0.0		8.1	2.96		72.0	>1200	>600	<10	<20	<100	>10
2017	0.00	238.2	20.9	8.3	2.79	54.8	48.2	0.8	16.9	4.7	48.9	2627.4	0.0
2017	1.00	236.6	21.1	8.3	2.79								
2017	1.50	237.2	20.8	8.3	2.79	54.5	48.9	0.8	13.0	4.2	54.5	2685.6	0.0
2017	2.00	236.6	21.0	8.3	2.79								
2017	3.00	235.8	20.7	8.3	2.79								
2017	4.00	234.7	20.9	8.3	2.79								
2017	4.50	234.5	20.5	8.3	2.78	53.8	49.8	1.0	17.2	4.5	55.1	2601.0	0.0
2017	5.00	224.6	20.2	8.3	2.78								
2017	5.50	195.9	19.6	8.3	2.79	55.8		0.8	16.5	4.3	52.3	2690.9	0.0
2017	5.75	213.5	19.7	8.3	2.79								
2017	6.00	135.4	18.4	8.3	2.80								
2017	6.50	93.7	17.4	8.2	2.80	52.8	48.1	0.6	25.4	3.5	35.8	2606.3	0.0
2017	7.00	59.1	16.8	8.2	2.80								
2017	7.25	48.1	16.3	8.1	2.80								
2017	7.50	16.5	15.8	8.1	2.80	54.3	49.5	0.7	31.3	2.6	22.5	2475.8	0.0
2017	7.75	11.9	15.6	8.1	1.80	38.4	38.6	1.3	33.2	2.7	21.3	2559.5	0.0
2017	8.00	1.0	15.1	8.1	2.80	50.3	48.9	3.1	34.0	2.3	14.7	2601.0	0.0
2017	8.50	0.7	14.9	8.0	2.80								
2017	9.00	0.1	14.5	8.0	2.80		52.1						
2017	9.50	0.0	14.1	8.0	2.80								
2017	9.75	0.0	14.3	8.0	2.81		49.7	28.9	40.2	0.8	0.0	2579.8	0.0
2017	(FFT)−0.08	0.0		7.8	3.08		75.0	>1200	>600	<10	<20	<100	>10
2018	0.00	251.6	20.9	8.3	2.53	32.5	32.0	0.3	16.4	1.2	0.0	2056.0	0.0
2018	1.50	247.5	20.0	8.3	2.48	48.9	47.5	0.4	24.9	1.3	0.0	2064.8	0.0
2018	4.00	227.8	18.2	8.2	2.41								
2018	4.50	214.4	17.6	8.2	2.34	48.6	44.9	0.4	21.7	1.4	0.0	2045.4	0.0
2018	5.50	211.3	17.4	8.2	2.33								
2018	6.00	206.3	17.3	8.2	2.33								
2018	6.50	191.9	17.0	8.2	2.32								
2018	7.00	181.6	16.8	8.2	2.31								
2018	7.50	125.6	16.1	8.1	2.29	37.1	35.8	0.3	27.2	1.3	0.0	2096.6	0.0
2018	7.75	68.8	15.3	8.1	2.27								
2018	8.00	35.9	14.9	8.0	2.22								
2018	8.50	0.3	13.4	8.0	2.20								
2018	8.75	0.0	13.2	8.0	2.19	51.6	49.4	6.4	41.5	1.0	0.0	2096.6	0.0
2018	9.00	0.0	13.1	7.9	2.19								
2018	9.50	0.0	13.0	7.9	2.18			6.1					
2018	9.75	0.0	12.9	7.9	2.18	52.2	43.0	13.6	34.6	0.9	0.0	2075.4	4.4
2018	10.00	0.0	12.8	7.9	2.17								
2018	10.50	0.0	12.4	7.9	2.16	52.2	48.5	26.9	28.6	0.9	0.0	2029.6	9.0

**Table 2:** Annual trends from August 2016 to August 2018 in the BML carbon pool in the entire water cap as well as in the hypolimnion and in epilimnetic oxygen concentrations (TC_F_, total carbon filtered (0.45 µm); TC_U_, total carbon unfiltered; TOC, total organic carbon; DOC, dissolved organic carbon; (av) H_2_O cap, averaged (av) value over entire depth profile; (av) hypolimnion, averaged value over hypolimnetic zone (>7.5 m); (av) epilimnion O_2_, averaged value of 0–4 m of BML water cap). Changes in year-to-year (∆) concentrations of these carbon pools, as well as epilimnetic oxygen concentrations as concentration and % are also shown.

	TC_F_	TC_U_	TOC	DOC	TOC/TC_U_	DOC/TC_F_
	mg L^−1^	mg L^−1^	mg L^−1^	mg L^−1^	%	%
	(av) H_2_O cap		(av) H_2_O cap		(av) H_2_O cap	
2016	214	219.8	72.2	68.5	32	32
2017	182.5	179.3	51.2	54.2	28	29
2018	172.5	176.1	45	43	25	26
	(av) hypolimnion		(av) hypolimnion		(av) hypolimnion	
2016	224.9	231.4	77.8	73.2	34	34
2017	181.9	162.2	47.7	46.7	29	26
2018	180.6	185.7	46.3	44.2	25	26
	∆ TC_F_ (%)	∆ TC_U_ (%)	∆ TOC (%)	∆ DOC (%)	∆ TOC/TC_U_ (%)	∆ DOC/TC_F_ (%)
	∆ (av) H_2_O cap decrease					
2016–2017	15	18	29	21	13	9
2017–2018	5	2	12	21	11	10
2016–2018	24	25	60	59	28	23
	∆ (av) hypolimnion decrease					
2016–2017	19	30	39	36	15	24
2017–2018	1	−14	3	5	14	0
2016–2018	5	25	68	66	36	31
	∆ (av) epilimnetic (O_2_) increase					
	∆ (O_2_)	∆ (O_2_)	∆ (O_2_)			
	μM	mg L^−1^	%			
2016–2018	24	0.75	11			
2016–2018	13	0.41	5			
2016–2018	37	1.16	17			

## Data Availability

Water column and FFT 16S rRNA gene amplicon sequences are deposited at NCBI under Bioproject PRJNA552483.

## Supplementary Materials

The following are available online at https://www.mdpi.com/article/10.3390/microorganisms10030510/s1, Figure S1: Time series of changes in light penetration (Secchi disk depths). Figure S2: Total periphyton biomass for BML (28-day colonization experiment at peak boreal summer). Table S1: Organic phosphorus for BML. Table S2: Amplicon sequence variants diversity.
