# Supplementary material for: Alum Addition Triggers Hypoxia in an Engineered Pit Lake
Source: Microorganisms. 2022 Feb 26;10(3):510. doi: 10.3390/microorganisms10030510 (PMC8953953; doi:10.3390/microorganisms10030510)
Supplement: Supplementary file 1 [file microorganisms-10-00510-s001.zip › microorganisms-1573342-supplementary.pdf]

Supplementary materials

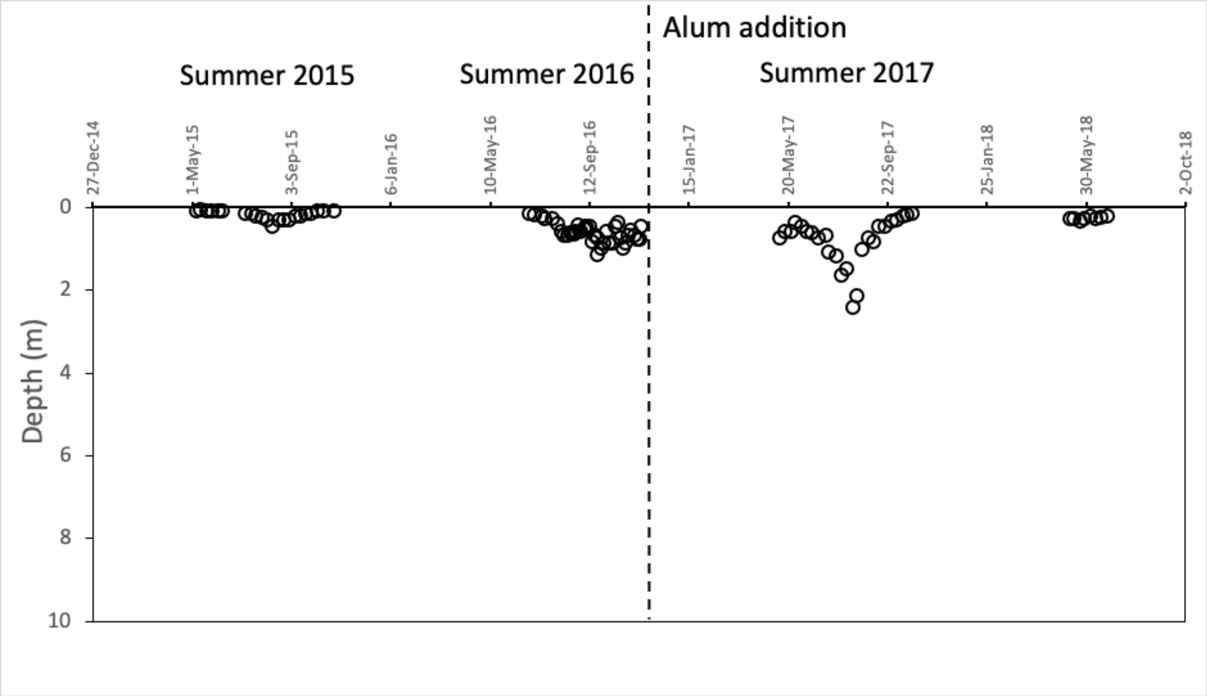

Figure S1. Time series of changes in light penetration (Secchi disk depths). Data from HATFIELD monitoring Program.

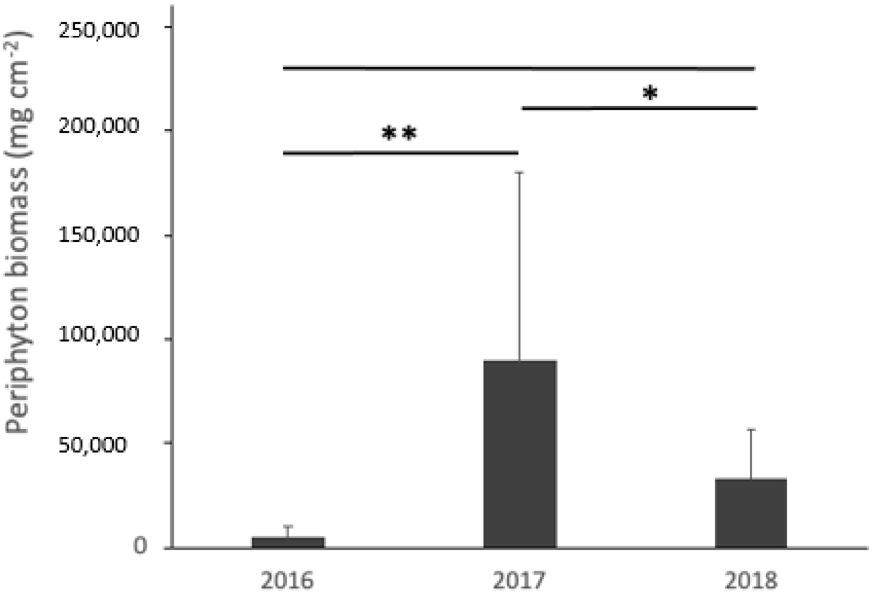

Figure S2. Averages (+S.D.) of total periphyton biomass for BML (28-day colonization experiment at peak summer) showing differences in biomass between years (ANOVA F (2, 33) = 7.68, p <0.01, Tukey Post-hoc Test, * p-value < 0.05, ** p-value <0.01). Data from and methods from HATFIELD monitoring Program. Periphyton colonization and growth rates were monitored on artificial substrates. Frosted glass slides (7.60 × 2.54 × 0.10 cm) were placed in floating plastic trays and deployed approximately 0.15 m below the water surface and secured to the platform in the highest sunlight-receiving orientation. Two floating trays were deployed and four glass slides were added to each tray at one-week intervals (e.g., July 31, August 7, 14, and 21, 2018); each floating tray held 16 glass slides at the culmination of sampling. Biovolume (μm^3) of each alga was estimated from the average organism dimensions and related to geometric shapes using a 630× magnification factor. The average biovolume of each cell was converted to biomass for all individual periphyton taxa assuming a specific gravity of 1 (i.e., 1 μm^3 = 1 µg). Total sample biomass (wet weight) for each taxon was calculated using the equation: Total Biomass ($\mu g/cm^2$) = Average Biomass (µg/cell) × Total Abundance ($cells/cm^2$).

Table S1. Organic phosphorus for BML. Data from HATFIELD monitoring program.

**Depth (m)**	**Porg (mg L^{-1}) 2016**	**Porg (mg L^{-1}) 2017**
0.3	0.0020	0.0020
2	0.0020	0.0020
4	0.0020	0.0020
6	0.0025	0.0022
8	0.0020	0.0020
9	0.0026	
9.75		0.0039

Table S2. Richness (based on ASVs) and diversity (inverse Simpson) for BML.

	ASVs			inv Simpson		
Depth (m)	2016	2017	2018	2016	2017	2018
0	218	300	287	45.8	16.6	18.6
1.5	361	352	326	49.8	14.4	24.8
4.5	253	429	263	44.0	16.3	32.7
5.5	221	402		47.2	16.3	
6	228			18.2		
6.5	476	470		32.2	23.9	
7.5		417			16.9	
7.75		428			30.1	
8 – 8.75		476	525		33.6	20.0
9.3-9.75	348	556	497	32.5	27.4	11.4
10.5			359			6.6
